# The lived experiences of relatives of autistic adults, and their perceptions of their relationships with autistic adults across multiple age-related transitions and demands: A qualitative interview study with reflexive thematic analysis

**DOI:** 10.1371/journal.pone.0294232

**Published:** 2024-01-19

**Authors:** Jahnese Hamilton, Tracy Finch, Ann Le Couteur, Joan Mackintosh, Alex Petrou, Deborah Garland, Jeremy R. Parr

**Affiliations:** 1 Research and Development, Cumbria, Northumberland, Tyne and Wear NHS Foundation Trust, Newcastle upon Tyne, United Kingdom; 2 Faculty of Medical Sciences, Newcastle University, Newcastle upon Tyne, United Kingdom; 3 Department of Nursing, Midwifery & Health, Northumbria University, Newcastle upon Tyne, United Kingdom; 4 Specialised Training Team, National Autistic Society, North East England, United Kingdom; University of Warsaw, POLAND

## Abstract

**Background:**

There is a need to better understand autism across the life course, including the lives of both autistic people and supporting relatives. As part of a larger mixed methods cohort study involving autistic adults, carers and relatives this sub-study focused on the experiences of relatives alone to learn more about the lives of people from the wider personal networks. Our research questions were: 1. What are the experiences of family members who care for and/or support autistic adults, 2. How can the viewpoints of relatives add to what we know about transitions and challenges experienced by autistic adults, and 3. What strategies/support have been helpful for adults and relatives?

**Methods:**

Relatives of autistic adults were purposively sampled and recruited using the Relatives/Carers cohort from the Adult Autism Spectrum Cohort—UK. 18 participants aged 31-81years who were related to 16 autistic adults aged 18-57years were interviewed for 24-91minutes. Interview transcripts were examined using reflexive thematic analysis.

**Main findings:**

Two overarching themes were developed, ‘Family support goes a long way in caring for autistic adults’ and ‘When families turn to society for support’ with subthemes. Relatives described benefits they had gained and their admiration for autistic adults. They reflected on how they gave support for independence in various contexts of dependence. They also identified the challenges that both autistic adults *and* families face navigating support systems (for example for healthcare and employment). An important novel outcome was the advocated value of role-models with lived experience who come from outside of the family.

**Recommendations:**

The findings lead to recommendations for: (i) Strategies to reduce the barriers for support that are faced by autistic individuals and relatives during crisis points; (ii) recognition and support for what enables both relatives and autistic adults to function independently (e.g. funded activities, flexible employment); (iii) future planning conversations to include relatives who can enhance knowledge and help plan for future care or support needs for autistic adults and (iv) opportunities for role models (persons with lived experience, autistic adults and relatives) to inspire others and disseminate knowledge.

**Conclusions:**

These findings add valuable insights into the experiences of relatives of autistic adults and challenge the reader to have greater appreciation of the many roles relatives can contribute across time and in a variety of contexts. These perspectives add important information for those working with and planning provision for autistic adults.

## Note on terminology

‘Autism’ is used in this article to cover all autism spectrum conditions, recognising name changes over time. Autism in this research manuscript includes Autism, Autism Spectrum Disorder, Asperger’s Syndrome, Atypical Autism, Pervasive Developmental Disorder-Not Otherwise Specified. We use the term ‘Autistic’ as this has been reported acceptable to many autistic adults, though some people may have other preferences [1].

‘Learning disability’ is the term used widely in the UK to describe heterogeneous conditions with childhood onset whereby there is significant impairment in functioning along with lower intellectual ability (usually IQ of less than 70). Internationally the term Intellectual Disabilities, and Intellectual Developmental Disorders are also commonly used. We use the term learning disability as this is the commonly accepted term in both UK Health and Social Care organisations and third sector organisations [2, 3].

## Introduction

Autism is a lifelong developmental condition that can impact on adult outcomes and is characterised by a profile of skills and needs, including challenges with social communication and social interaction [4, 5]. The experiences of autistic adults reported in the published literature frequently highlight the low levels of social participation, limited number of friendships [6], and just how few autistic adults are in paid employment [7]. Studies focusing on health reveal high levels of both mental and physical health problems [8–10] and lower levels of service access [11, 12], all of which can impact poorly on quality of life [13]. Around 40% of autistic adults have co-occurring learning disability [14], which may mean the individualised provision may require a commitment to more open-ended support interventions alongside more structured and specified care arrangements than individuals without learning disability [15]. Autistic adults with and without a learning disability can find aspects of independent living and the communication of their needs and experiences to be challenging. Involving the network of individuals around a person can be one way of gaining insight into their lived experiences, from the viewpoints of those who know them well.

Qualitative research has given some autistic adults the opportunity to reflect on their lives. In our parallel study, Finch and colleagues interviewed 29 autistic adults across the adult life span to understand experiences of autism throughout adulthood [16], and in other literature Hickey and colleagues report interviewing 13 autistic adults who were aged over 50 years old about their experiences of getting older [4]. These in-depth accounts give autistic adults the opportunity to provide first-hand accounts of their lived experiences and highlight a variety of life experiences and reflections on adulthood. These accounts bring a mixture of pride and/or disappointment in achievements e.g., in education or employment. Commonly these reflections also indicated a sense of difference from others, a drive to seek connection with others, exhaustion from trying to conform to social norms, value in autism support groups, and frustration with the inaccessibility of adequate healthcare and practical support. While some autistic adults reflected that living with others in a relationship or cohabiting sometimes reduced loneliness, there were mixed reports of both supportive and unsupportive relationships with parents. Frequently the picture appeared to be of individuals battling on alone in adulthood and later life, and this raises questions about how involved the families of autistic adults are in adulthood, what are relatives’ perspectives, and what ranges of experiences are reported by relatives who care for and/or support autistic adults with varying ranges of ability?

Research with relatives has looked at the within family relationships. Parents have described a sense of developing personal competence, pride in their autistic adult relation’s progression, and an increased appreciation of others [17], as well as high levels of stress frequently focused on worries about autistic adults’ futures [18–20]. Findings have highlighted the support relatives give adults in the tasks and activities of daily living regularly, or at specific times of need [21], and the steps relatives go to in order to provide security and take responsibility for caring throughout their lives, e.g. by reducing their employment hours and income [22, 23]. Relatives can be central to gaining knowledge of rights, finding out what support can be accessed and facilitating service involvement [24], and akin to the experiences of autistic adults, relatives can also often experience heightened levels of social isolation themselves [6, 20]. Further research on relatives ‘coping’ skills, recognises personal resources, strong caring relationships with autistic individuals, and spiritual and social networks to be important sources of support [25–27].

Understanding more from the individuals who are close to autistic adults, and their combined development and experiences over the life course is crucial for learning more about the wider determinants of the health of both parties from a social support and access perspective, and for considering how support should be provided. This is more than a question of insight into the lives of autistic adults themselves. Research suggests that ignoring or failing to give weight to these close relationships of importance may increase the risk of poor transitional development for autistic adults [28]. If this is the case then it is essential that we understand more about the wider personal network too, holding in mind health and wellbeing for both autistic individuals and the network of informal carers in their families on whom they rely. Qualitative research can explore experiences, enhance knowledge and identify new topics and research questions of importance [29]. This study aimed to interview relatives to gain new information and understanding about relatives and their experiences caring for and supporting autistic individuals with a variety of needs in adulthood. Autistic adults’ first hand experiences were collected and reported separately [16]. This qualitative study provides the opportunity for relatives to share their experiences. Our broad research questions are, 1. What are the experiences of family members who care for and/or support autistic adults, 2. How can the viewpoints of relatives add to what we know about transitions and challenges experienced by autistic adults, and 3. What strategies/support have been helpful for adults and relatives.

## Methods

### Community involvement

This research was developed to address research priority areas identified by autistic adults, relatives, clinicians, and researchers at a community involvement event [30]. Autistic adults and relatives of autistic adults co-developed and piloted the topic guide and materials for the study including the invitation letter, participant information booklet and consent forms. Autistic adults also reviewed the write up of the manuscript for wording sensitivity and representation.

### Participant recruitment

Relatives of autistic adults were selected using a nonprobability non-random purposive sampling strategy [31] targeting relatives of autistic adults of different ages from the Relatives/carers cohort of the Adult Autism Spectrum Cohort–UK (ASC-UK) [30] (https://research.ncl.ac.uk/adultautismspectrum). Eligibility criteria were: Participant in ASC-UK; self-identified as a relative of an autistic adult; able to communicate in English; willing to be interviewed; within travelling distance of the North East of England in case face to face interview was preferred by the participant. There were no exclusion criteria. Sampling continued in batches of four until the interviewer and research analysis team assessed there to be enough information to report a wide range of experience i.e. theoretical sufficiency in the data [32]. Theoretical sufficiency rather than data saturation was selected for pragmatic reasons because of time and financial constraints.

### Materials

A topic guide (see S1 File. Relatives interview topic guide) for the qualitative interview was co-developed, piloted and refined with autistic adults and relatives of autistic adults. The guide contained brief closed and open questions to encourage discussion around caring for and supporting autistic adults and what had gone well and not so well. Questions began with context setting, the relationship type, age of autistic relation and their age at diagnosis and other diagnoses/health difficulties. The experience of gaining an autism diagnosis and having a diagnosis was explored from the perspective of the relative, and their opinion sought on how this was experienced by their autistic relation. Questions explored relatives’ own lives and asked about the impact of caring on their own physical and mental health, personal relationships, living arrangements and employment. Relatives were also asked whether or not they had received social support. The topic guide gave the interviewer a broad question to ask in each area and then suggested more focused areas for prompting as appropriate. Demographic and background information was collected by self-report questionnaire at ASC-UK registration. Other materials co-designed for the study included the invitation letter, participant information booklet (see S2 File. Participant information booklet), and consent form (see S3 File. Informed consent form).

### Procedure

The study methods have been checked against the Standards for Reporting of Qualitative Research (SRQR) Checklist is provided (S1 Checklist). NHS Research Ethical permissions were obtained from Wales Research Ethics Committee 5 (REC reference: 14/WA/1066). Potential participants were recruited to the Cohort element of the study and then a sub-sample was contacted for the Qualitative study in 2016 and 2017. Contact was made using their preferred method of communication (email, postal letter, or telephone). Participants were given information about the study in advance. This included a general overview of the study, and well as the topic guide questions (prior to the interview) and an opportunity to ask questions before giving voluntary informed written consent for interview. Participants were invited to take part using their preferred interview format. These options included to meet for interview face to face or if they preferred by telephone or video conferencing, alternative formats such as using email or video diaries were also offered for participant preference. Participants were informed that participation was voluntary, and they were free to not answer questions or to end the interview at any time if they chose. Informed consent was documented and signed for using NHS Research Ethics authorised consent forms. Interviews took place at the participant’s preferred location, their homes or university premises. Partners were permitted to take part in the interview with the primary participant providing they gave informed written consent. No autistic adults were included in these interviews to allow focus on relatives’ views. Autistic adults from a separate sample with no relationship to this sample were interviewed in the parallel study, reported separately [16]. No incentives were paid for participation. One member of the research team who is a trained qualitative interviewer conducted all the interviews (JM). Each interview was flexibly adapted to suit the communication style of the participant whilst following the topic guide probes to explore the interviewee’s views.

Only authorised members of the research team who required access to personal details for the purpose of carrying out the research had access to information that could identify individual participants during or after data collection. Confidentiality of the interviews was maintained by the interviewer and recordings were deleted after being sent for transcription. Transcripts were anonymised prior to data analysis. Therefore, only the qualitative interviewer (JM) was able to identify participants from their data and additional measures were taken to protect the anonymity of participants at each data processing phase: Each interview was audio recorded using a participant ID number, transcribed by a confidential transcription company, and anonymised using a pseudonym in preparation for analysis. All transcripts were checked by JM for completeness and anonymised to remove any potentially identifying information prior to analysis. Data from couples interviewed together were included in analysis, but to ensure successful anonymisation, the information was reported in the write-up using the pseudonym of the first participant only.

### Analysis

We undertook a Reflexive Thematic Analysis [33, 34] to qualitatively examine and interpret the data while reflecting on our subjectivity and orientation to the analysis. The analysis aimed to be inductive to prioritise the data itself for the identification of the themes rather than use a pre-existing theoretical frame, as we wanted a bottom-up influence on the findings. We also wanted to personally and collectively reflect on what we might see more of due to our prior experiences and make an effort to question this and to look for what might potentially be unseen [35]. The aim was to keep an open mind with a focus on what exactly the participants themselves were trying to communicate.

Taking on an essentialist/realist epistemology we considered the language used to directly communicate experiences and appraised the data content at a semantic level by regarding the words as explicit communication. We recognised our drive to hear what the interviewees wanted to say and were motivated to give empathic accounts of this. We were also conscious of possible influence from our personal and professional backgrounds and sought to manage this by asking ourselves questions on why we were drawn to certain elements and whether other elements were also being given appropriate consideration.

The research analysis team (JH, TF, and JM) conducted the analysis. This was an iterative approach where the researchers discussed the interviews and the transcripts and considered codes and potential theme ideas from first exposure and over time. This began with familiarisation, included an immersive period of comprehensive coding and a process of interpretation and elaboration of the theme definitions and their relationships over time.

The interview transcripts were read, discussed, reread, provisionally coded and potential themes considered. The initial codes and their relationship to different (sometimes multiple) tentative themes were noted across the transcripts and listed (see S4 File. Code and theme development, Tables 1 and 2). The research analysis team were able to reflect upon what had been heard in interview (JM) and read in the transcripts (JH, TF and JM), and challenge and debate each-others interpretation of the data. Since the members of the research analysis team had clinical and research experience but no personal experience of caring for an individual with autism or learning disability, they took an outside perspective on the interview content. Individually and collectively the team worked to be aware of their individual interpretations, possible influence from personal backgrounds and attention to related research projects across the wider research programme.

**Table 1:** Relatives (participants) pseudonym and information on the autistic adult relation.

Participant pseudonym	Autistic adult relation age band, sex (M/F); with/without learning disability
**Andrew**	20–24 (M); with learning disability
**Theresa**	20–24 (M); with learning disability
**Eleanor**	25–29 (M); with learning disability
**Harriet**	30–34 (F); with learning disability
**Naomi**	55–59 (M); with learning disability
**Jenny**	18–19 (M); without learning disability
**Irene**	18–19 (M); without learning disability
**Denise**	20–24 (M); without learning disability
**Debbie**	20–24 (M); without learning disability
**Lesley**	20–24 (M); without learning disability
**Caroline**	20–24 (M); without learning disability
**Stanley**	25–29 (M); without learning disability
**Yvonne**	30–34 (F); without learning disability
**Rosemary**	35–39 (M); without learning disability
**Pauline**	35–39 (F); without learning disability
**William**	55–59 (M); without learning disability

**Table 2:** Aggregated data on relatives and autistic adults’ relationship type, demographic and health information.

Relatives (n18)		Autistic Adults (n16)	
Age (years)	31–81 (median 59.5)	Age (years)	18–57 (median 24.5)
Gender: Female	13	Gender: Female	3
Gender: Male	5	Gender: Male	13
Ethnicity	17 White (one unknown)	Age at autism diagnosis	3–51 (median 17)
		Learning disability	5
**Relationship to Autistic Adult:**		**Living situation:**	
Parent	14	With wife and children	1
Grandparent	2	With parents/ grandparents	8
Spouse	1	Independent	5
Sibling	1	Supported living placements	2
**Relationship status:**		**Relationship status:**	
Married / in a long-term relationship	14	Married	1
Widowed	1	Previous romantic relationships	3
Divorced	2	Single	12
Single	1		
**Education (highest attainment):**		**Education (highest attainment):**	
University level education	6	University level education	4
A-levels or equivalent	4	A-levels or equivalent	0
Basic school qualification / GCSEs or equivalent	4	Basic school qualification / GCSEs or equivalent	8
No formal qualification	2 (two unknown)	No formal qualification	4
**Employment status:**		**Employment status:**	
Retired	9	Employed	2
Carer to family member	3	Intermittent employment–currently unemployed	4
Full-time employed	3	Unemployed	2
Part-time employed	3	Full time student (mainstream courses)	2
		Student (specialist autism courses)	3
		None, attends day care centres	2 (one unknown)
**Health status:**		**Health status:**	
Physical health conditions	8	Physical health conditions	5
Mental health conditions	3	Mental health conditions	13
No current health problems	8 (two unknown)	Epilepsy	4
		No current health problems	1
		Speech and communication difficulties	5
		Dyspraxia/mobility problems	4

Using an initial code list developed inductively, each transcript was comprehensively coded sentence by sentence by JH through careful reading of the data with consideration to the context of the responses in relation to the questions posed. Within this process the codes list and themes were renamed as appropriate to give a greater accuracy in their description of the data. More codes were added when there was no good fit with the existing codes. Notes were made about relationships between data and codes when data connected to more than one code and/or related to more than one theme. The names of the themes were expanded and tentative sub-themes began to be considered. The comprehensive coding was then reviewed and reflected upon by TF to give a second opinion on the coding decisions and theme development. Again, this process involved personal and joint introspection on our own identity and professional backgrounds (e.g. being female, bringing up children, having received education at university level, other research projects we were involved in), and how this might influence our interpretations [34, 35].

Themes were then re-considered for their scope and split or regrouped as required to best represent the coded data they connected to (see S4 File. Overarching themes that revealed a super-structure and connections between themes were identified and named. Underlying themes and their sub-themes were considered together with the coded data and mapped out (see S4 File). This process was led by JH and reviewed by TF.

The findings were then written up with reflective quotes selected to illustrate. This was reviewed and reflected upon by the lead author with the research team, (TF, ALC, JP) leading to the insertion and trimming of some quotes for brevity of illustration or to remove potentially person identifiable information.

### Participant details

Seventeen relatives were invited to participate. From these invitations, 16 individuals plus two of their spouses gave informed consent and were interviewed, opting for face-to-face interviews usually in their homes, occasionally at the university. Interviews lasted 24-91minutes. The relatives were related to 16 autistic adults across the working age adulthood age-span, five of whom had learning disability (see Table 1). Aggregated data summarising relationship with autistic adults, demographic details and health information for both relatives and autistic adults are presented in Table 2. The metadata is also provided in S5 File. Descriptive data metadata.

#### Relatives’ demographics (see Table 2)

The 18 participants (five males and 13 females) were of White British ethnicity, and aged 31–81 years (median age 59.5). Fourteen relatives were married/in a long-term relationship, one was widowed, two divorced and one single. They were of mixed socioeconomic status as shown by qualifications and occupation: Highest level of qualifications were, six Higher (University) Education, four had A- levels/NVQ level 3/equivalents, four had GCSE/O-level/NVQ level 2/equivalent, and two had no formal qualifications. For employment, nine were retired, three described themselves as carers for family, three worked part-time in admin/secretarial or assistant roles, one full time in sales, one full-time management and one self-employed.

In terms of personal health, eight relatives reported physical health conditions and of these three also reported anxiety/stress/depression. Eight had no current health problems (two ‘unknown’). No relatives reported having an autism spectrum diagnosis.

#### Information about *autistic adults* (see Table 2)

The relatives (above) were related to 16 autistic adults (13 males and three females; aged 18–57 years: reported in Table 1 within age bands to conceal identity). Nine autistic adults lived with their relative, five lived independently with varying levels of family input, and two had supported living placements. Most autistic adults were single. One autistic adult was married and three had been in romantic relationships at some time.

Autistic adults’ occupations included two currently in employment, two undertaking full time mainstream studies, six were unemployed but of these four had intermittent employment previously, three were on specialist autism courses placements, and two attended day care centres (one ‘missing data’). Highest qualifications were, four had Higher (University) Education, four had GCSE/O-level/NVQ level 2/equivalent, four had basic school qualifications, and four had no formal qualifications.

All except one of the autistic adults were reported to have additional current physical health and/or mental health conditions, five had coexisting learning disability. The autistic adults received a range of autism diagnoses (Autism/Autism Spectrum Disorder/Asperger’s/Atypical Autism) made at different ages, six were diagnosed in childhood, two in adolescence, three in early adulthood before they were 30 years old, and five in later adulthood between 30–55 years old.

## Results

### Analytical themes

**Fig 1:**
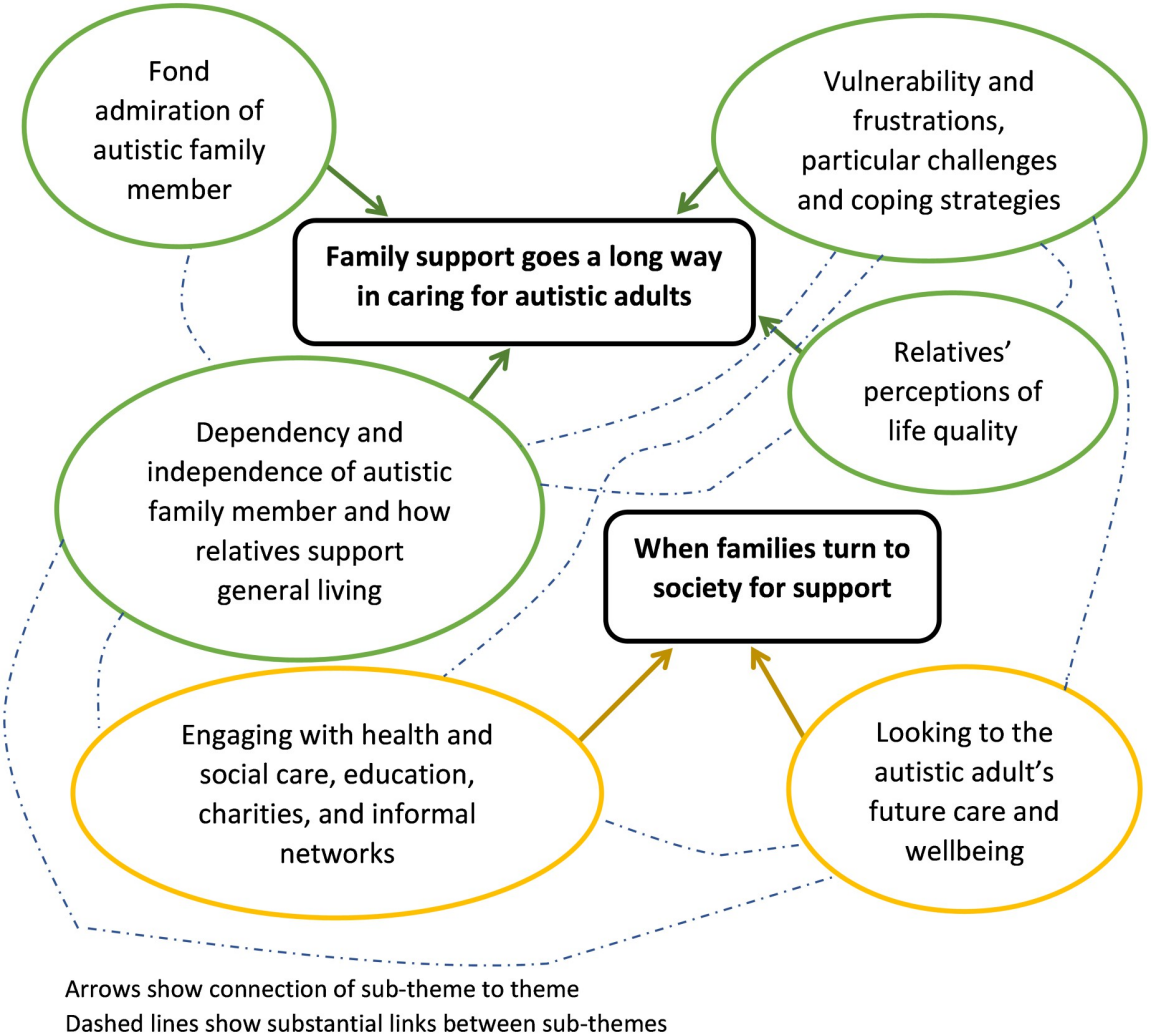
Themes with corresponding sub-themes and relationship links.

The thematic analysis of the interview transcripts identified multiple levels of themes during the initial iterations of theme development (see S4 File. Code and theme development, Fig 1 for an example). These were reviewed and refined within four iterations into the final two overarching themes: ‘Family support goes a long way in caring for autistic adults’ and ‘When families turn to society for support’ with six sub-themes and substantial links between these where codes connected to multiple themes, representing the many relationships between (Fig 1).

Coded data related to one or more sub-theme within an overarching theme, with some of the data relating to more than one thematic area. Where codes related to more than one theme this was considered evidence of relationships between themes (see Fig 1).

Within each theme were additional groupings or an additional layer of sub-theme showing further level of organisation of the codes. An example of this and how the codes fitted with theme layers is shown in Fig 1 within S4 File. Code and theme development, which shows the example of overarching theme 1 ‘Family support goes a long way caring for autistic adults’ and the sub-theme ‘Vulnerability and frustrations, particular challenges and coping strategies’.

The inductive approach to the analysis meant that the resulting themes reflected what relatives wanted to communicate to others when asked about areas of their lives in relation to caring for autistic adults. These overarching themes and their content provide insights into the experiences of family members who care for and/or support autistic adults and into the challenges faced by autistic adults, at different stages of life for both autistic adults and for the relatives themselves. These accounts reveal much about what was understood to be held within the family, what experiences were being encountered in different domains of life when interacting with others, and what strategies/support have been helpful for adults and relatives.

### Theme 1: Family support goes a long way in caring for autistic adults

Relatives reported strong feelings of affection and allegiance, often within contexts fraught with frequent tensions. Relatives lived experience reflected the lives and experiences they share with autistic adults. Relatives’ perspectives reflected on personal feelings of positivity, experiences of overwhelmingly challenging times and of success or relief in overcoming challenges over time, and of recognising positive results from the support they have provided. Relatives recognised autistic adults’ strengths as well as their vulnerabilities. There was a strong sense of personal responsibility held by relatives, which tipped into concern about autistic adults who possibly in the future would no longer have family support due to relatives ageing and loss.

#### Sub-theme: ‘Fond admiration of autistic family member’

Relatives spoke warmly about good times in family life and enjoying being together, ‘*we’ve been good for each other’* (William) and having fun together over the many years of their lives, ‘*he’s got a fantastic sense of humour*. *He’s keenly intelligent*. *He’s very amusing*. *He can see things in a very different way*. *But he just can’t seem to make those connections’* (Jenny).

Relatives recognised different achievements autistic adults had made from attaining employment, living independently or with formal/informal supported living, learning new skills, driving, performing, singing, dancing, caring for horses, helping with cleaning, through to coping with aspects of daily life despite constant challenge, ‘*I admire him quite a lot*, *how he does cope*. *I think it must be frustrating for him*, *not being able to speak’* (Theresa).

Some of the stand-out joys came from activities made possible through autism specific provision or funding,

*[He] is a very talented [musician]*, *and part of his independent budget*… *They pay for him to go to […] Its band*… *all the young people have autism*, *and they are absolutely amazing […] Absolutely amazing […] They’re travelling up and down the country*, *doing gigs*, *twice*, *sometimes three times a week*. *Abroad next year*

(Irene)

Be it at home or as part of a group, self-funded or with supported resource, it was clear that in all the time together they had shared, relatives had built a lifetime wealth of proud memories.

#### Sub-theme: ‘Dependency and independence of autistic family member and how relatives support general living’

Related to ‘fond admiration’, is the sub-theme of dependency and independence. Relatives share much of their lives with autistic adults, supporting living generally, supporting transitions and growth, and being there to help cope with adverse circumstances. Relatives recognised how an adults’ autism was making them vulnerable, and the investment needed from family to help autistic adults fight for their rights. For example, when they had missed out on financial benefits, faced obstacles in trying to access healthcare, felt unable to defend their employment, or didn’t have enough money to pay for food. In these situations, relatives had knowledge of what autistic adults could achieve if they overcame the obstacle presented,

*I went with him*, *most interviews*, *because I felt like I had to fight his case because he wouldn’t push himself; he’ll just take it at face-value*, *what people tell him and won’t question it*, *whereas I will*. *I just thought*, *you know what*, *he was treated like people that had been unemployed for years and he had a really good track record of working*

(Naomi)

Considering Naomi’s account it is possible that her relative would have become stuck in an experience of joblessness had she not supported him through the next stage, and is one example of the important role relatives may take in supporting autistic adults through many year-on-year life transitions across adulthood.

Relatives spoke of supporting their autistic relative to get back on their feet after a crisis, access health services, clear debts when not having enough money or having given money away, and access support for living. Relatives also acknowledged how their own strong finances and the power to influence were relied upon to help achieve support for autistic adults,

*the education psychologist*, *[…] solicitor […] It cost a lot of money*. *[…] if you didn’t have those means*, *basically forget it [or] ability to cope with this […]*. *Everything is an uphill struggle*, *and everything is a fight and everything wears you down*

(Andrew)

Andrew was reflecting on a particularly intense period of transitioning in early adulthood, supporting their autistic adult relation (who also had diagnosed learning disability) into supported independent living outside of the family home.

Some more able autistic adults moved out of the parental home for work or higher education. Relatives spoke of their contributions to make this possible, e.g., through providing financial support,

*He works in a […] office*, *taking the bookings*, *but he only does about 15 hours*. *He gets very stressed if he works full-time*, *[…] We bought the flat for him*, *[…] we scraped some money together and got the flat for him*, *just to know he was in a safe area*

(Rosemary)

Rosemary’s autistic relation was in his late 30’s, did not have a learning disability, but his autistic needs meant that while he could manage some steady employment he could not take on the number of hours or amount of responsibilities required to generate enough income to cover all of his living costs, notably appropriate housing.

For autistic adults with greater dependence needs, with and without diagnosed learning disabilities, relatives described different indicators of independence. This could be the ability to self-occupy for a period, visit the cinema with friends, or developing enough familiarity with places to be able to walk around alone safely,

*We’ve got a hotel in [country] that we’ve been taking him to since he came to live with us […] He can wander around*. *He talks to people there*. […] *He goes to the shops just 100 yards up the road*. *He spends hours talking to the staff because they haven’t changed*

(Irene)

Some autistic adults required 24-hour care, ‘*it is just like having a small child*. *You know*, *like showering somebody every day*. *I’ve bathed somebody everyday practically for 40 years*. *Do you know what I mean*?*’* (Harriet). Harriet’s autistic adult daughter with learning disability was in her early 30’s but her needs were reflected on as that of a young child. With the lived experience of knowing what support autistic adults needed, albeit at different levels of intensity, relatives relayed the difficulties of what had been. This was also tied to worry about future care, showing the relationship between this sub-theme considering dependency and independence and sub-themes of the second later theme of when families turn to society for support.

Looking within the family network, some relatives reported that questions arose amongst family members, including siblings, as to who would be next to take on more intense caring roles, ‘*I can’t imagine her ever being able to live independently and that’s something that really*, *really worries me’* (Yvonne). Yvonne’s autistic relation was in her early 30’s, did not have a learning disability, but her needs made it unlikely she would ever move out of the family home, though which and whether there would be family members able to care for her across her lifespan was a worry on the mind of the family.

Parents wanted other offspring to be concerned for the welfare of their autistic sibling, but not to have the full-time caring role. Relatives spoke of the impact of caring on their own relationships with spouses, wider family and subsequent constraints on life choices. For some families, difficulties had led to siblings moving out to live elsewhere. Careers were tailored to fit with caring duties, some had given up paid employment. Couples described taking shift-type turns in caring, and how this made them feel,

*I’ll tell you what I noticed the other day, I saw a couple retired and you could see they were probably early sixties, and they were walking down the street hand in hand and I thought, I’ll never do that because I’ll always have him in tow with me*

(Eleanor)

Eleanor’s autistic adult son was in his late 20’s and diagnosed with cooccurring learning disability. She didn’t think there would be any days in her week where she wasn’t directly caring for him. These reflections relate to other sub-themes of recognising vulnerability, frustrations, and coping, and of life quality discussed further below.

#### Sub-theme: ‘Vulnerability and frustrations, particular challenges and coping strategies’

Relatives were concerned about autistic adults’ vulnerability. They gave examples of what had happened in the past and could happen again, such as lending out money that was not repaid, not eating, not wearing adequate clothing to stay warm, or living in particularly vulnerable circumstances,

*he tells me he used to do drugs when he was up here in the children’s home […] then gets into this bad company*. *He starts injecting drugs*, *the pins as they’re called apparently*, *and he ended up having [a serious infection]*

(Stanley)

Substantial accounts of autistic adults’ mental health crises were given. Sometimes these were triggered by bullying, adverse life circumstances such as losing employment, or stress trying to fit in. Suicide attempts with significant and lasting harm, severe self-neglect and hospitalisation were described, along with long recovery times with much support needed and given by relatives,

*she loved […] learning […] But it was too intense*. *It was very*, *very stressful and she just crashed out*. *She crashed out big style*. *She has major depression*. *[…] She is still finding her feet because she was admitted in [hospital]*, *it must be three years ago when she crashed out from the hospital […] She was quite poorly*. *But she is definitely improving*. *She is definitely picking up and this diagnosis [of ASD] has made a really big difference*. *We will just wait and see how it goes*

(Pauline)

Pauline’s autistic adult relation appeared to manage intellectual demands of adulthood well, entering higher education and training for employment. However, in the context of reportedly finding the learning experience to be too intense, experienced a mental health crisis with enduring mental health needs. This experience also triggered access to an autism diagnosis in adulthood that Pauline hoped would then be helpful in terms of contextualising the whole experience and assist with understanding herself and her needs better in future years.

Some relatives were exposed to behaviours from autistic adults that they found difficult to manage and some accounts relayed crisis points where urgent assistance from emergency services had been called upon to help manage risk to themselves, the autistic adult or property. Challenging behaviours, perhaps manageable with a young child, were now problematic due to the strength of the adult,

*I think her autism makes her more difficult in an environment*. *I think it makes her really anxious and really panicky and she doesn’t like being in with lots of people and if she sees strange environments*, *she panics and she just totally… And*, *she’s really strong*. *She can run around a room*, *and you can’t hold her down to do anything and they won’t now*. *[…] when [Adult] kicks off*, *it can be scary*. *It’s terrifying for me so for people who don’t understand it is [terrifying]*

(Harriet)

Continuing difficulties with behaviours that challenge made some relatives anxious being out in public, ‘*I worry a lot more now than I would when I was younger […] now I’m*, *like*, *“They probably think I’m a bad mam*, *and they don’t understand*,*” and that sort of thing’* (Caroline). Caroline’s autistic son was now in his early 20’s, without learning disability, but with ongoing behaviour that is challenging for the family to manage in public places.

There was also frustration with others (including family members) who didn’t understand autism, ‘*[his] own father still won’t accept the diagnosis*, *and he still thinks he’s just spoilt’* (Jenny), and needing to explain the autistic adult’s capabilities and needs for assistance, ‘*[they] will say to me*, *Has he got a good memory*? *I say*, *Actually no’* (Andrew).

Two mothers spoke of ‘blaming themselves’ for their adult son’s autism or difficulties with social interaction; searching for imagined ‘causal’ reasons: genetics, alcohol during pregnancy, their own personality, ‘*I thought that if I was more social then he would be able to make more friends’* (Jenny).

There was often an intense sense of relatives feeling they were on the borderline of coping/not-coping in their caring role, and yet a belief that without their input their adult relation would suffer immensely from the inaction of others or lack of support structures in society,

*If you’re not equipped to do it and if you can’t handle it emotionally*, *first of all you’ll get nothing*. *The child will get nothing […] I’m still fighting and looking after my son despite the fact that there’s allegedly a provider there*

(Andrew)

In this case, which was reflected in other accounts too, the diagnosis of autism even with co-occurring learning disability did not grant smooth access to appropriate social care support, even after a care package had been agreed.

Relatives also described coping over long periods of time and overcoming crisis points. These accounts revealed a wealth of expertise held by relatives, for example helping autistic adults use strategies to help manage sensory needs, aid relaxation, support healthy lifestyles, manage schedules or social expectations, and develop independence skills, and to de-escalate situations,

*When he goes*, *he goes and you just have to ride with it*. *I tend to take my eye contact away from him*, *take language away from him and he comes down and you can see him coming down*. *[…] You just have to take the language away and he’ll come down himself and try not to put him in situations where he’s going to get anxious*. *That’s the best bit*. *Try and anticipate it before it happens*

(Eleanor)

With this realisation of coping, and of crises passing, and of autistic adults getting back on their feet, many of these discussions linked to the subsequent sub-theme of life quality.

#### Sub-theme: ‘Relatives’ perceptions of life quality’

This sub-theme considered both the perceptions of experiences of the autistic adult and relatives’ own lives. Focusing on the lives of autistic adults, relatives considered employment, engagement in hobbies (physical activities and musical interests), and the ability to share these achievements socially in the company of others or in performances as indicators of quality of life. They described the benefit of assessing their autistic family member’s quality of life through a lens of what can be, instead of what may be lost, ‘*what I would say*. *I think it’s important for people*, *when they’ve had a diagnosis*, *to know that it’s not the end*. *[*…*] They do have a life*. [Adult]*’s one of the good results’* (Lesley). Lesley’s autistic adult relation had entered early adulthood with relative ease, and his family recognised he had a good quality of life.

Relatives also considered their own quality of life. The impact of responding to crises, supporting general living, and the long-term impact of daily (and/or nightly) stressors was thought by some relatives to affect their own physical and mental health, ‘*he doesn’t sleep at night’* (Debbie; living with autistic relation in his early 20’s who doesn’t have a learning disability). However often health problems in relatives were considered part of ageing, not as a result of having caring roles, but this also added to concerns about the future, *‘my hips are going*, *I’ve got problems with my knees’* (Irene). Just over half of relatives who participated in this study had physical or mental health problems themselves.

Overall relatives were keen to share what was working well in their lives, what made them happy, and their achievements over time. This included engaging in personal interests and hobbies when alone and with friends and being able to take on work or pursue educational opportunities. Despite worries about the future care of autistic adults, relatives recognised positives and described being optimistic about the successes they had experienced,

*My work works*, *you know*. *My home life works*. *My daughter’s happy […] And [autistic adult]’s passed his driving test*, *with the hope of more freedom*. *And I think I feel really quite optimistic about where we are at the moment*

(Denise).

Denise’s autistic adult son was in his early 20’s, without a learning disability. He had experienced mental health crises which were difficult times for the family, but having got through these Denise felt positive about his achievements and about his future.

### Theme 2: When families turn to society for support

Relatives often invested much in supporting autistic adults, and only turned to society with particular social and/or health needs at particular times. They reported mixed outcomes and a sense of many barriers to overcome for both autistic adults and for themselves as they attempted to advocate. When reporting experiences of support (including from both formal and informal networks), relatives invariably considered the future and what help their autistic family members would receive. Many of these conversations showed how stressful the topic of asking for support was. Recognising and reflecting on these experiences may be useful to services and organisations who aim to support autistic adults, with and without coexisting learning disability and their carers.

#### Sub-theme: ‘Engaging with health and social care, education, charities, and informal networks’

This large topic focussed on the experiences of accessing or attempting to access support for needs beyond what the families’ own resources could manage. Relatives recognised support that was needed either now or in the future, ‘*we’ll have to think about some long-term care*. *She’s got a normal life expectancy’* (Harriet), along with support for the relatives who care to be able to continue in this role.

*If we can have the respite*, *we’re able to keep [adult] at home and surely that’s more beneficial than having to go into a permanent placement*? *[…] Maybe years ago*, *I didn’t need as much respite*, *but I think now I do*. *I feel like I need those nights just for a break really*

(Harriet)

Support was valued from a variety of sources including respite arrangements, autism charities, social groups, helplines and peer supporters, and funded autism specific social and arts activities. Health and social care staff who were considerate and sympathetic, recognised idiosyncratic needs and made reasonable adjustments [such as a named doctor or a quiet waiting space] were especially appreciated. Autism diagnostic services were often pivotal in helping relatives understand autism and enabling access to autism services or charities. Informal organised networks set up by families to support each other e.g., coffee mornings or online forums were also highly valued, ‘*what I’ve found over the years is that the information you get […]*, *it normally comes from other parents who are ahead of you in the cycle’* (Andrew).

Positive recognition was given to staff who could see autistic adults as individuals, ‘*she picked up that he has got a good sense of humour*, *but it was a one-way sense of humour’* (Naomi). This quote illustrates the importance and lasting impact of good personal interactions between families and the different professionals and agencies.

There was however an overwhelming sense of insurmountable barriers to accessing suitable, timely, and consistent support for autistic adults and relatives. The examples included access to services providing short breaks (referred to as ‘respite’ above), mental health, autism diagnosis particularly for adults with a learning disability; support for independent living; and advice on where to turn. Funding issues included access to health and social care (with some reporting that accessing funding for epilepsy or learning disabilities was substantially easier than for autism), and inadequately funded services, ‘*social workers were constantly*, *I felt*, *working the budget’ (Eleanor)*.

Relatives spoke of the extreme efforts it took to get help, the absolute absence of support in their localities, and of how draining this apparently endless task was on their emotional resources, ‘*I feel badly let down by the system’* (Rosemary). As a relative, Rosemary saw first-hand that support was needed to enable independent living, but also experienced that this support needed to come from family members as it was not adequately provided from social support organisations.

At its worst relatives felt criticised by staff in services when they did ask for help and felt prevented from advocating needs in the notion of ‘adult autonomy’,

*When he plunged back down again*, *[date] it was horrendous*, *[adult] wouldn’t leave the house*. *I tried to get him help*, *by ringing the mental health team*, *but they wouldn’t take a referral from me because he was an adult*

(Denise)

There was an expectation in healthcare that autistic adults would take themselves to doctor appointments or access services independently for help, even though it was known they had additional needs. In Denise’s case her son was under 25 years old and living as a dependent in the family home.

A particular lack of services for autistic adults was noted in the areas of community support, community activities, and vocational activities or adjusted employment opportunities for autistic adults who were unable to access mainstream provision. Autism charities, though highly valued, were often not equipped to help with long term needs,

*I signed up to the local autism society*, *and as part of that sign-up somebody came round*. *You got a couple of sessions with somebody*. *And they mentioned Personal Independence Payment*. *[…] It was nice being able to talk to this lady who came round*, *because her son was autistic*, *and she was able to share her experiences*. *So it was helpful*. *But then it was just a couple of sessions and that was it*

(Jenny)

In summary accessing support was at times successful, yet many barriers existed. A particular obstacle was the belief that services should not engage with relatives as this jeopardised the autonomy of the autistic adult. This predicament was frustrating for relatives trying to manage on their own with autistic adults who they believed were not receiving appropriate care. Further many services are time limited, requiring repeated applications, and only available after relatives’ intensive advocacy, exacerbating worries about the future.

#### Sub-theme: ‘Looking to the autistic adult’s future care and wellbeing’

Relatives had many suggestions for how autistic adults could be better supported, as well as how other families could be helped across the life course. One suggestion was for there to be greater access to role models with lived experience,

*This person knew somebody who was at […] University who was doing a […] degree*. *So we connected them*. *I think it was great for [him] to see that you could be autistic but still have a life*, *and still contribute to society*, *and still achieve something*. *So I think having that kind of buddy system […] I think talking to other people who have come out the other side*

(Jenny)

People with lived experience sharing that experience explicitly or implicitly enabled a sense of hope, and perhaps instilled ambition for what could be attained.

Another suggestion was for autistic adults to be given the choice to nominate preferred personal contacts as their advocate when dealing with organisations, ‘*the people who have got the problem should be able to nominate people to give them support*, *because basically they’re a vulnerable adult’* (Stanley). The nominated advocate proposal was considered a possible mechanism by which relatives or friends who provided care and support informally could more easily engage with services designed to offer formal support.

Reflecting on support from services, relatives also suggested that greater awareness of autistic adults’ needs could potentially be attained through recruiting more staff with personal experience of autism. High quality autism training for all staff was also recommended.

Other practical suggestions were for the development of case manager roles to coordinate communication and applications for assistance across different organisations including both health and social care as well as financial, benefit, and tax organisations.

Suggestions for future wellbeing also considered opportunities that could be met by the business sector or non-governmental organisations,

*There’s nothing like a directory*. *If you like cookery*, *there’s something here*. *If you like dancing*, *there’s something*. *If you like music*, *there’s something there*. *If you like art*, *there’s something there*. *There’s nothing*. *You tend to*, *when something does come about*, *it tends to be parents setting stuff up*. *There’s a massive gap in the market*, *you know*? *A massive gap*. *I keep saying this*, *for people opening businesses for stuff like this*, *especially out of hours stuff*. *There’s a massive gap*

(Eleanor)

This account vocalised an urgent need for high quality evening and weekend care and activities, that relatives would potentially pay for, if they had the funds. For Eleanor whose autistic adult relation requires daily care, it was important for her to consider both time-out for her own needs, as well as consider how to provide a variety of pursuits for her adult son to engage in.

In supporting relatives to support adults, relatives also endorsed the value of linking with other people with personal experience of caring and recommended the development of mentoring systems or network groups to achieve this. Relatives also suggested carer specific support such as counselling and a carers-crisis helpline to support relatives to be able to do their best in the care they give.

In looking to a future time when potentially the relatives are no longer here, relatives thought about future security, ‘*the one thing that I need to do is I need to make sure that when I’m gone at least he’s got a house where he can feel safe and secure’* (Jenny).

Relatives expressed hope that having an autism diagnosis might help future access to appropriate support, ‘*she might get better support because of having the diagnosis’* (Harriet). In this Harriet recognised that different ways of helping her middle-aged autistic adult daughter came from understanding autism, above and beyond recognising her daughter’s needs coming from her learning disability. With gaining the co-occurring autism diagnosis, Harriet was more hopeful for appropriate support being provided in the future. However, relatives also spoke of being unsure of what would happen in the future lives of autistic adults and at times fearful. Ageing and loss were a reality for many families, with death of grandparents and parents contributing to an inevitable reduction in support networks, ‘*I’ve still got my mum and dad*, *and they’re elderly now […] he’s nearly 80*, *and she’s 75 […] but I can see a black hole coming*. *I know it’s coming*. *I don’t know what happens then’* (Theresa). Theresa’s son was only in his early 20’s, he has co-occurring learning disability and she could see their joint support network dwindling. Specific concerns relatives had included whether autistic adults might experience neglect or self-neglect, e.g., through not being encouraged to eat well or maintain personal hygiene, or be subject to other forms of abuse, or simply not really cared for, ‘*nobody’ll care for [him] like I’ll care for him’* (Theresa). For autistic adults not involved with services, relatives anticipated risk of poor health and destitution,

*In the longer term*, *I’ve got severe concerns about whether he could live on his own*, *because he can’t manage money […] Because I think there’s no doubt that*, *had he not had me*, *in the period where he was*, *kind of*, *not going to Jobseekers*, *he would have ended up homeless […] The things like having a home and clothes and food are not of any importance to [him]*. *They’re just not*. *They’re not a priority*

(Denise)

Denise’s adult autistic son did not have a learning disability, however Denise’s lived experience allowed her to understand her son’s vulnerabilities. The relationship between thoughts and anticipations in this theme is closely connected with experiences described in the earlier themes about vulnerability, dependency, and engagement with services.

In summary, relatives in this study were definite about the importance of considering themselves ‘in the picture’. They emphasised that alongside recognising the needs and skills of autistic adults, services (especially health and social care) should work collaboratively with relatives acknowledging their caring role and responsibilities.

## Discussion

This qualitative study sought to learn from the experiences of family members who care for autistic adults, questioned how the viewpoints of relatives might add to what we know about transitions and challenges experienced by autistic adults, and asked what strategies or support has been helpful for adults and relatives. The findings provide valuable insights on autism in adulthood from the perspectives of other family members/relatives, exploring both relatives’ impressions of their autistic adult relation and their own experiences. Relatives highlight the wide range of autistic adults’ achievements and needs, and how relatives’ value their relationships with the autistic adults. Alongside these positive experiences relatives described the complex range of interactions with services and networks to gain advice and support over time and their concerns about the impact of ageing.

Perhaps surprisingly participants acknowledged that this was the first time they had been given the opportunity to tell their own stories. The relatives wanted to communicate how much support and crisis management they are involved in across the life course. They shared their experiences of what worked well and not well when interacting with services and were keen to say how things could be improved.

Relatives are frequently the people closest to autistic adults as they may have lived together through the adult’s development, or as a partner/spouse during adult life. In keeping with contemporary studies relatives spoke of recognising the positives of caring in relation to their own personal growth [36] and valuing the closeness in their relationships with autistic adults [17, 37]. Relatives appreciated how emotionally challenging it is for autistic adults across a wide range of activities expected in adulthood, and this is important for the reader and the wider community to hold in mind: trying to find work, succeed in education, organise finances, and communicate their needs and experiences while frequently coping with autism specific challenges such as sensory overload [38] and communication difficulties, and also coping with other coexisting disabilities [8–10] is not an easy feat, and these views mirror the accounts given by autistic adults themselves [16, 39].

Relatives often saw their role as helping autistic adults get the most out of potential opportunities (getting back to work, seeing their healthcare team, organising accommodation, or taking a holiday together), which suggests the family itself can be an important source of support for adults. While there were many reports of positive interactions with a range of services and individual professionals, relatives consistently described the exhaustion associated with the ‘ongoing fight’ to access specialist support. Particularly when in situations where there was a lack of understanding about the advocacy role relatives were trying to take on to ensure the needs and rights of autistic adults were met. Considered in relation to research with autistic adults on their experiences of service access [39–41], the current analysis is striking, highlighting that both autistic adults and relatives report facing barriers to securing appropriate care for autistic adults. This emphasises that services should address the potential barriers affecting both autistic adults and their advocates.

In keeping with other studies, this study highlights the needs of relatives providing informal care, particularly as both parties age. Relatives confirmed the physical, financial, and emotional toll on themselves from providing support, advocacy and practical care throughout their lifetimes and into older age [20, 21, 23]. It was notable that just two autistic adults had supported living placements with more than half still living as dependents in the family home. With dwindling informal support networks as relatives’ extended families also aged and passed away, and a lack of longitudinal service involvement, it is clear why relatives worry about their autistic relative’s future and the family’s ability to provide for these needs [19].

Despite these concerns, in the ‘here and now’ relatives reflected on how themselves, other family members and autistic adults had often worked together to create lifestyles that worked for them. They had developed interaction strategies that worked for autistic adults, found flexible employment that enabled co-caring roles, and overcome intense crises. Many recognised how times had improved and acknowledged their joint ability with autistic adults to overcome challenges together as long as they are present and well enough.

In considering what other strategies/support have been helpful for adults and relatives or could be better, there were repeated accounts about the benefit of contact with role models: Role models for autistic adults who are other autistic individuals, and role models for relatives who are other relatives. This may not be mutually exclusive however as autistic supporters of autistic relatives may also be potential role models. Examples reported were an autistic adult going to university and speaking to a younger autistic friend, and accounts of relatives benefiting from being in contact with other people who also had life experience of caring for autistic adults. This highlighted the suggestion that the greatest support came from those with lived experience (*ahead of you in the cycle*). This is different to previous research which points to the benefit of role models within the family [42, 43]. In this study relatives highlighted value in role models with; (a) lived experience, and (b) who come from outside of the family. The benefits and potential benefits emerging from visible inclusion of individuals with lived experience, as role-models for both relatives and autistic adults, is an area deserving of further research and utilisation in the strategic development of service provision.

### Strengths and limitations

A strength of this study is the focus on the experiences of relatives of different ages and from a range of socioeconomic backgrounds supporting autistic adults of differing ages and abilities (with and without coexisting learning disability). In the interviews the relatives reflected on their own lived experiences during different life stages, as they supported an autistic adult relative who might not necessarily easily communicate their own experiences. It is a strength that the voices of relatives added to previous literature on the roles of the wider family and how families cope over time, and also their shared needs (often to be included and listened to). It is also a strength to hear the benefit of learning from others with lived experience, and the advocacy for more visibility of individuals with lived experience in positions where they can offer support and inspiration.

There are limitations associated with the decision to interview only relatives of autistic adults. While the study allowed relatives an opportunity to ‘feel heard’ and provide a voice for those autistic adults perhaps unable to speak for themselves, this study did not include the autistic adults that the relatives were discussing. We have conducted a parallel qualitative study of experiences across the lifespan with a separate sample of autistic adults [16]. However, none of those individuals were the autistic adults discussed in this study. Future studies may benefit from adopting an inclusive approach where different family members, relatives and autistic adults, contribute together, or through a series of interviews to understand the perspectives of a range of interviewees.

There are also other limitations in the sample selected. Although the sample size was appropriate for a qualitative study and included relatives across a wide age range and socioeconomic and educational background, a definite limitation was the absence of representation from people of Black, Asian, or other minority ethnic backgrounds. It is also notable that the proportion of male participants was low, 5 male relatives in comparison to 13 female relatives participated and two of those came in as partners in the interviews rather than via direct recruitment. Future research should consider how to encourage male relatives to have more of a voice. In contract the autistic relations had a small representation of female autistic adults, 3 females to 13 males, which again could be considered in future studies. In addition, participants were mainly parents or grandparents and were recruited from or within travelling distance of the North East of England. Further research to compare experiences from relatives from other ethnic groups and from wider geographic representation would be important to extend these findings.

Finally, it is noted that while this study included relatives and autistic adults in the design of the study and in the write-up, which is a strength, it did not include any member checking within the analysis, which is a limitation. Qualitative research that seeks to give representation to community voice may benefit from greater validation during the analysis stage by member checking or other incorporation of participants within the active process of analysis.

### Implications for research and practice

1. An overwhelming message from the current study is the importance of investigating ways in which services might work more effectively with autistic adults *and* their relatives and carers. This applies both at crisis points and proactively to promote health and wellbeing.
2. Relatives also emphasised several areas that potentially benefit autistic adults and relatives to function independently to the best of their ability, including available finances for example to access hobbies/artistic pursuits/training, strength of advocacy voice, flexible working, supportive family, and for some, paid carers and respite.
3. Research is needed to investigate how the perspectives of different ‘experts by experience’ such as persons with lived experience of autism and relatives of autistic individuals, can be incorporated to promote best practice and improve service provision.
4. Lived experience was greatly valued for insight and leadership. Opportunities to learn from those who have walked the path ahead can be maximised to facilitate planning conversations with relatives about current and future care, support, or opportunities, potentially within training programmes for empowerment [24] tailored to different transitions across the life course.

## Conclusions

Relatives can provide a unique perspective on the lives of autistic adults perhaps especially where direct communication with some autistic adults may be difficult [44] or potentially intrusive [45]. Taken alongside the experiences of autistic adults [4, 16, 39], these insights offer breadth of views, including consideration of positive experiences, constructive solutions, and appreciation of need across different situations, transitions and crisis points.

In this study, relatives were rarely defined as formal carers but frequently had some responsibility for the welfare of their autistic adult relative. Recognising a need for flexibility and collaboration when working with autistic adults and relatives is an important yet complex task. Autistic adults and relatives with a range of different lived experiences serve as important role models for others in their learning and sense of what might be achieved. Service providers and wider society can gain new insights and perspectives when the contributions of people with lived experience are acknowledged in supportive and educative roles.

## Supporting information

S1 File Relatives interview topic guide. (PDF)

S2 File Participant information booklet. (PDF)

S3 File Informed consent form. (PDF)

S4 File Code and theme development. (PDF)

S5 File Descriptive data metadata. (PDF)

S1 Checklist Standards for Reporting Qualitative Research (SRQR) checklist. (PDF)
