# Supplementary material for: The lived experiences of relatives of autistic adults, and their perceptions of their relationships with autistic adults across multiple age-related transitions and demands: A qualitative interview study with reflexive thematic analysis
Source: PLoS One. 2024 Jan 19;19(1):e0294232. doi: 10.1371/journal.pone.0294232 (PMC10798545; doi:10.1371/journal.pone.0294232)
Supplement: S1 File — (PDF) [file pone.0294232.s001.pdf]

QUALITATIVE INTERVIEW TOPIC GUIDE – RELATIVES OF ADULTS ON THE AUTISM SPECTRUM

1. Introduce researcher and purpose of the study
2. Obtain consent to proceed and to record the conversation
3. Remind interviewee that all information remains confidential, and that they are free to stop the interview and withdraw at any time.
4. Check that interviewee is comfortable and happy to proceed

Adult relative with Autism – context setting

- In what way are you related to the adult with autism?
- How old is the adult?
- What is their diagnosis?
- How old were they when diagnosed?
- Does your relative have any other difficulties, in addition to their autism diagnosis?

Diagnosis

Age at diagnosis – Has your relative received a formal diagnosis? If so, how old were they?

- How did the diagnosis come about?
- What was the process of diagnosis like for you? What was good about it, and what was less good?
- (FOR THOSE DIAGNOSED AS ADULTS) What was the process of diagnosis like for your relative?
- How has your life been affected by the diagnosis?
  - How has the diagnosis been useful?
  - Are there ways the diagnosis has not been useful and caused difficulties for you?

Post diagnosis

- What information did your relative receive after their diagnosis?
- What about you – what information did you receive after your relative's diagnosis?
- What support did you receive?
- Do you think you'd have preferred more information about a certain topic? If so, which topic?

- What support services do you think would be useful for relatives of adults after diagnosis? How should these be provided?

Relatives' everyday lives – question for <u>all</u>

Please could you tell us something about your own everyday life?

- What aspects of life go well for you nowadays? What is more difficult?
- Looking back, what has gone well for you over the years? And was there a period when things went less well? Why do you think that was? Looking back, do you think you needed more support? In what way? What might have been useful to you?
- How satisfied with your life are you, in relation to your goals, your expectations, and things that concern you?

Physical Health

- Has caring for/supporting your relative with autism affected your physical health?
    - If so, how?
        - What has been difficult for you?
        - What would have made it better?

Mental Health

- Has caring for/supporting your relative with autism affected your mental health?
    - If so, in what ways?
        - What has been difficult for you
        - What would have made it easier?

Personal relationships

Do you feel that caring for/supporting your relative with autism has affected your own personal relationships with other people – for example with family members, or partners? If so, how?

- What has gone well with relationships?
- What has not gone so well?

Do you feel that caring/supporting your relative with autism has affected your relationship with each other?

- If so, in what ways?

Living arrangements

Do you think that caring for/supporting your relative with autism has affected your living arrangements? If so, in what ways?

Then additional questions about:

- Housing
- Financial support
- Support from family

Social support

Do you think you have had enough social support to help you to care for/support your relative with autism?

What support has been important? Was there a time that was particularly useful? Has there been a time support was missing? If so, when, and what would have been more useful to you?

Additional questions might focus on

- Family
- Friends
- Health professionals
  Social care professionals
- Services/organisations/agencies

Employment

Has having a relative on the autism spectrum affected the kind of work you do? (incl. paid, voluntary, domestic etc) If so, in what ways?

- What has made it difficult for you?
- What would have made it easier?
- Was there any support available to help you?

That is great – thank you. Can we just spend the last 5 minutes or so talking about the things you yourself like to do?

Thank you very much. At the end of the study we will let you have a summary of what we have found and of course the study team will keep in touch with you about the XXX study.
