# Supplementary material for: The lived experiences of relatives of autistic adults, and their perceptions of their relationships with autistic adults across multiple age-related transitions and demands: A qualitative interview study with reflexive thematic analysis
Source: PLoS One. 2024 Jan 19;19(1):e0294232. doi: 10.1371/journal.pone.0294232 (PMC10798545; doi:10.1371/journal.pone.0294232)
Supplement: S2 File — (PDF) [file pone.0294232.s002.pdf]

Hearing about the views and perspectives of relatives of adults on the autism spectrum

This booklet contains information about a further piece of research that you may be interested in.

Please read the information inside. If you would like to take part, please complete the consent form and send it back to us.
Thank you

Autism Spectrum, Adulthood and Ageing

Hearing the views and perspectives of relatives of autistic adults

Hello. My name is XXX. I work at XXX University. [Insert Photo]
Thank you for taking part in the project 'Learning about the lives of relatives of adults on the autism spectrum'. We are inviting some of those taking part to discuss with us in further detail issues that we know are important to relatives of adults on the autism spectrum. We would like to have more in depth discussions as it is not always possible to find out what people think from answers on questionnaires.

If you agree to take part, we will ask you to meet or speak with us, or communicate in another way about [INSERT TOPIC]. This is an opportunity for you to tell someone about the issues that are important to you as the relative of an adult on the autism spectrum. If you take part, we would like to give you a £20 voucher as a gesture of thanks. We will cover any travel costs.

What are the aims of this part of the research?

- To collect further information from relatives of adults on the autism spectrum. This will help researchers, policy makers and those providing services for relatives and adults to better understand their skills and support needs
- To understand in more detail how life experiences change over time for relatives of adults on the autism spectrum, and for adults themselves
- To answer important questions which may lead to significant advances in knowledge, opportunities and care

Why have I been invited to take part?

We are inviting you to take part in a discussion because you have completed a questionnaire as part of the Relative's study being run by XXX University; we would now like to hear from you in more detail.

How might this discussion help me and other relatives of adults on the autism spectrum?

There may be no personal benefit to yourself. However, some people welcome the chance to talk about certain topics, including life experiences for your relative with autism and yourself – for instance, what has gone well, and what has not gone so well. By talking about these important aspects of your lives you will help us to understand how to improve services and support for you, or for other people. The research team includes adults on the autism spectrum, and parents/carers who are

advising us about the best way to collect information needed to better understand people's everyday lives.

Do I have to take part?

No. It is up to you to decide whether or not to take part in a discussion. The research team are available to talk about the discussion with you if you wish, and our contact details are at the end of this information sheet.

What happens if I don't want to continue?

You may withdraw at any time and this will not affect services you may access now or in the future.

What do I have to do?

If you agree to take part we will ask you to sign a consent form. After we receive your signed consent form, a researcher from the university will contact you.

If I agree to take part, what will happen next?

You will be asked to participate in a discussion in the way that suits you best. For instance, we could meet you to have a discussion or if you are comfortable in discussion group, you could join a small group session. We can have a discussion by telephone or using email, skype or video diaries. You may be able to suggest other ways for us to communicate. With your permission, our discussions will be audio-recorded, although we won't do that if you don't want us to – you could still take part.

The researcher will ask a range of questions about your experiences – we would send you a copy of the questions beforehand so you are aware of the topics for discussion

If you would like to meet up, you can choose where this would be. We can provide a meeting room at the university, or if you prefer, we can meet at your own home or other local place, e.g. a café or library – we would need to find a quiet, and fairly private place.

We want you to feel at ease and relaxed during the discussion. Before it takes place, we'll ask you what things we can do to make the discussion as easy as possible for you. For instance, you might want someone else to be present as a supporter, or someone to meet you afterward. Relatives of adults on the autism spectrum are working with us on the research, and have let us know their thoughts on how we can help the discussions be as easy as possible. If you do take part, we would like to give you a £20 voucher as a gesture of thanks, and we will cover any travel costs.

Will my information be kept safe and confidential?

After the discussion, your information will be kept safe according to the Data Protection Act 1998. Your name and contact details will only appear on the consent form and this will be stored separately from the electronic data files and any typed copies of the discussion details. The written copies will be read only by members of the research team and the person who writes them. Any recordings will be deleted after they have been transferred to the computer and checked. Once checked, the written summaries will be anonymised. Only members of the research team will be able to look at these summaries that will be labelled with a number only. Your personal details will not be used. In any published information, no identifying details will be attached to any quotations.

What will happen to the results of the research study?

We will let you know the results of our studies by sending you a summary. We will also tell people about the findings through meetings, through our website and social media. We also intend to publish our findings in research summaries and scientific journals to ensure that they are read by people locally, in the rest of the UK and abroad. We hope that the results of the study will be used to improve the services that are available to adults on the autism spectrum.

Who is organising and funding this research study?

This study is being run by the autism research team at XXX University, working with groups from across the UK. The team includes experienced autism researchers, and researchers who are experts in studying adulthood and ageing, as well as adults on the autism spectrum. For a list of the people involved see the website www.autismspectrum-uk.com. The study is funded by the UK charity Autistica (www.autistica.org.uk).

Who has reviewed this research study?

This research has been looked at by an independent committee of people, as part of the National Research Ethics Standards (NRES) procedures. This process is designed to protect your interests and ensure all research is of a high standard. This study has been reviewed and given a favourable ethical opinion by Wales Research Ethic Committee 5 14/WA/1066. If at any time you want advice about taking part in this study, you can contact the Patient Advice and Liaison Service (PALS) in your local NHS Trust through the hospital switchboard (details are also on the Trust website).

Thank you for taking the time to read this Information Sheet. If you have any questions, please contact one of the researchers, see below:

Contact details: XXX, Tel. XXX, email XXX
