# Supplementary material for: The lived experiences of relatives of autistic adults, and their perceptions of their relationships with autistic adults across multiple age-related transitions and demands: A qualitative interview study with reflexive thematic analysis
Source: PLoS One. 2024 Jan 19;19(1):e0294232. doi: 10.1371/journal.pone.0294232 (PMC10798545; doi:10.1371/journal.pone.0294232)
Supplement: S3 File — (PDF) [file pone.0294232.s003.pdf]

For Office Use ID:

Supporting information 3. Informed consent form

Hearing about the views and perspectives of relatives of adults on the autism spectrum

Consent Form for relatives

Please **initial** or **mark** the box if you agree

Please read each of the following statements. **You do not have to agree with all of the points – please just mark the ones you do agree with.**

1. I have read and understood the **Hearing about the views and perspectives of relatives of adults on the autism spectrum** information sheet (version XX dated XX) and have had the opportunity to have any questions answered. ☐

2. I understand I am free to stop taking part in the interview study at any time. I understand that data collected up to that point will be anonymised and can still be included in the study, unless I request it be destroyed. ☐

3. I understand that my information may also be looked at by the agencies ensuring the quality of the research, including from XXX. I give permission for these agencies to have access to information that the research team collects, once my name has been removed. Otherwise, I understand that all identifying information will be kept confidential. ☐

4. I am willing to take part in a discussion interview with a researcher. ☐

5. I am willing to take part in a group discussion along with other relatives of adults on the autism spectrum. ☐

6. I understand that the discussion could take place in person or by telephone or through another method, and that I will be asked my preferred method when the researcher contacts me. ☐

7. I understand that anonymised information from this study may be studied in the future by other researchers undertaking relevant projects (with the prior agreement of the Research Committee). ☐

8. I agree to allow the researcher to audio-record the discussion. I understand that anything that can personally identify me will be removed from the typed transcripts of the interview, and that the transcripts will be used for research purposes ☐

9. I understand that once transcribed, audio-recordings will be destroyed and transcripts stored in locked files in accordance with the Data Protection Act. ☐

____________________________	____/____/____	______________________
Name of Participant	Date	Signature/Mark
____________________________	____/____/____	______________________
Name of Person taking consent	Date	Signature
