# Supplementary material for: The lived experiences of relatives of autistic adults, and their perceptions of their relationships with autistic adults across multiple age-related transitions and demands: A qualitative interview study with reflexive thematic analysis
Source: PLoS One. 2024 Jan 19;19(1):e0294232. doi: 10.1371/journal.pone.0294232 (PMC10798545; doi:10.1371/journal.pone.0294232)

Supporting information 4. Code and theme development

Contents

Coding 1
Table 1. Coding iterations of transcript data 1
Colour key for early themes grouping coding 2
Table 2. Codes, colour coded and presented in accordance with links with sub-themes 3
Theme development 5
Table 3. Theme development iterations and changes 5
Table 4. Thematic table showing over-arching Theme 1. 'Family support goes a long way for autistic adults' with four corresponding themes and eleven sub-themes below 8
Table 5. Thematic table showing over-arching Theme 2. 'When families recognise they are in need of support from society' with two corresponding themes and six sub-themes below 8
Example 9
Figure 1. An example of how an overarching theme and sub-theme linked, with additional underlying groupings and codes 9

Coding

Table 1. Coding iterations of transcript data

1st Iteration coding	2nd Iteration coding	3rd Iteration coding	4th Iteration coding
Read transcripts, consider, and discuss reflections with analysis team (including interviewer)	Reread transcripts, initial codes noted across transcripts, reread transcripts and adjust coding as necessary. Consider initial themes, Note initial codes and their	Compile a list of all of the codes noted in the transcripts. Use the code list to comprehensively code each transcript sentence by sentence with consideration to context in the discussions. Reflectively discuss codes list and themes with the	Redefine and rename as appropriate to improve accuracy and add codes when the existing codes do not fit. Note relationships between data and codes when they connect to more than one code and/or theme.

	relationship to early themes.	analysis team, with team reflection on full set of coded data.	

Colour key for early themes grouping coding

- *Fond Admiration*
- *Dependency and independence*
- *Vulnerability and Frustration*
- *Life quality*
- *Engaging with society, health and social care, education, charities*
- *Looking to the future*

Table 2. Codes, colour coded and presented in accordance with links with sub-themes

**Codes**	**Corresponding theme**
Relative valuing caring for adult (fond of), sense of purpose and how it has changed them as a person<br><br>(links with impacts of caring on relatives)	**Fond admiration**
Communication with other people misunderstanding autism /their acceptance with the diagnosis / or relatives self-acceptance with the diagnsosis	
Admiration of adults qualities by relative	
Achievements by adult	
Levels of independence in adulthood, living, financial, employment	**Dependency and independence**
Support needs of adult, communication, health, managing money<br><br>(links with vulnerabilities)	
Dependency of adult on relatives for health and wellbeing / for asserting and advocating on behalf of adult	
Advocacy to other agencies	
Encouragement to adult to motivate day to day and big achievements	
Contrasting childhood to adulthood for adult / aging changes / memories of childhood challenges	
Comparing childhood to adulthood caring needs and options to access	
Form of relationships adult has with others	
Impacts of caring on health, relationships, wellbeing of care giver	
Financial and employment impacts of caring	
Networks of support carers rely on and challenges in finding additional care for adult when needed	
Working arrangements of relative to fit with caring responsibilities	**Vulnerability and frustration**
Vulnerability of adult	
Financial vulnerability of adult	
Risky behaviour in adult	
Challenging behaviour, aggression, self harm, crisis points	

Other differences – other issues that interact with vulnerability?	
Relatives frustrations to do with challenges /overwhelmed	
Vulnerability of adult	
Strategies to support adults needs – formal vs informal? (eg medication a special issue?)	
Communication with other people misunderstanding autism	
Consideration, contrasts, with other family members, siblings , relatives frustrations to do with challenges due to competing demands	
Blame of self for child's autism	
Quality of life of adult	**Life quality**
Quality of life of relatives	
Adjustments to support capacity, to support inclusion	**Engaging with society, health and social care, education, charities**
Frustrations with communication with health and social care providers – staff turnover, not understanding the person and their needs	
Good communication/care from health and social care	
Barriers to accessing support, employment, health and social care, activities	
Experience of diagnosis and impact – relative's experience of the diagnostic process, delivery, support (or lack)	
Notion of 'adult autonomy' generating challenges	
Finding out and sharing information and support could be accessed / informal support networks	
Ideas for what would help if it could be provided, recommendations	**Looking to the future**
Fear for the future care of adult	
Aging care givers	

Theme development

Table 3. Theme development iterations and changes

1st Iteration Themes Identification from analysis team (4 themes)	2nd Iteration Themes Identification (5 themes)	3rd Iteration Themes Identification (6 themes and sub-themes)	4th Iteration Themes Identification (2 overarching themes, with sub-themes and sub-sub themes)
**Early steps in theme identification as emerging from the coded data:**<br><br>Initial Codes reordered into the four early themes. Rough code descriptors pulled from group theme charts from each independent qualitative analysis of text.<br>Green – JH,<br>Black – JM,<br>Blue - TF<br><br>THEME 1: **Fond Admiration**<br>Of Adults idiosyncratic Abilities<br>Closeness with adult<br>- Ways of realising self-worth/potential (attainment in education)<br><br>THEME 2: **Vulnerability**<br>Of the adult (education attainment, employment, social relationships, independent living, general support needs)<br>Of the support system provided by relatives – to adult and to each other, relatives own health and wellbeing and social support<br>Views from others, views of others	**Developing the naming of the emerging themes, reviewing relationships with codes and identifying an additional theme, looking at tentative relationships between these:**<br><br>***1. Fond Admiration*** *(overlaps with second). This is about the feelings the relative has for their autistic adult, what they see them doing well, achieving, what their qualities are.*<br><br>***2. Dependency and independence*** *(overlaps with all). This is about the relationship between the relative and the autistic adult, how independent the adult is, and how dependent they are on their relative for help with health, care, communication, finance, employment etc.*	**Recognising the groupings of codes and holding these groups together as sub-themes, with their relationships with themes. Recognising two themes previously grouped together as distinct from each other:**<br><br>***1. Fond Admiration***<br>• ***Love for autistic adult***<br>• ***Recognition of achievements***<br><br>***2. Dependency and independence***<br>• ***Independence of autistic adult***<br>• ***Care and advocacy needs***<br>• ***Impacts of caring on relatives***<br>• ***Supportive relationships***<br><br>***3. Vulnerability and Frustration***<br>• ***Autistic adult's vulnerabilities***<br>• ***Relatives' frustrations with challenges***<br>• ***Misunderstanding autism***	**Recognising the closeness of relationship between themes and with this the identification of two overarching themes:**<br><br>**1. Family support goes a long way caring for autistic adults**<br>• Fond admiration<br>• Dependency and independence<br>• Vulnerability and Frustration<br>• Life quality<br><br>**2. When families recognise they are in need of support from society**<br>• Engaging with health and social care, education, charities, and informal networks<br>• Looking to the future<br><br>**While the sub-themes drawn out in iteration 3 continue to be relevant reporting of three layers of themes was considered too complex to facilitate a clear overview of the findings, therefore**

- Prompting need for diagnosis, challenges with this, recognised with diagnosis<br>- "Invisibility" of HFA<br>- crisises and violence to self and others<br>- Crisis services often won't speak to parents of adults (data protection)<br>- Support to leave the house/socialise/work<br>- Quality of support depends on where you live<br>- Dependency and limitations charity-based services<br>- health and wellbeing<br>-<br><br>THEME 3: **Dependency**<br>Needs of adult for care and support from relatives for living and social experiences Advocacy – education, health, finance. aim of work to provide for adult child<br><br>For family - A life built around supporting the Adult with autism Coping Making best of life, Mourning for A's 'lost' life Recognised challenges and limitations in families, Grief, guilt and Anger Family – juggling the needs of different members e.g. others health and wellbeing, on social experiences of family Importance of financial resource Experiences of accessing services, support & resources	***3. Vulnerability and Frustration*** *(overlaps with second). This theme recognises the vulnerability of the adult, and the challenges they and the carer face. Risky and challenging behaviour and other differences are included here, and how this impacts on the adult, the relative, and others.*<br><br>***4. Engaging with society, health and social care, education, charities*** *(overlaps with second, third and fifth). This covers the experiences, good and bad, that that the relative and their adult have had engaging with services and organisations.*<br><br>***5. Life quality and Looking to the future*** *(overlaps with all). This captures elements of quality of life from both the adult and relatives, and also the anxieties relatives have when they think about the future, about their own aging as carers, what suggestions about what would help others.* (NOTE: CHANGE TO LOOKING TO THE FUTURE AND MOVE	***4. Engaging with society, health and social care, education, charities***<br>• ***Care providers strengths and weaknesses***<br>• ***Relatives' abilities influencing outcomes for autistic adult***<br>• ***Role of informal support networks***<br><br>***5. Life quality***<br>• ***Quality of life of autistic adult***<br>• ***Quality of life of relatives***<br><br>***6. Looking to the future***<br>• ***Aging caregivers***<br>• ***Fear for future wellbeing of adult***<br>• ***Relatives' suggestions for improvements***	**overarching themes and first layer underlying themes only are reported in results.**<br><br><br>**Relationships between underlying themes revisited with reference to codes:**<br>**1.** *Fond Admiration (overlaps with second)*<br>**2.** *Dependency and independence (overlaps with all)*<br>**3.** *Vulnerability and Frustration (overlaps with second, fourth, fifth and sixth)*<br>**4.** *Relatives' perceptions of Life quality (overlaps with second and third)*<br>**5.** *Engaging with society, health and social care, education, charities (overlaps with second, third and fifth)*<br>6. *Looking to the future (overlaps with second, third and fifth)*

Need for peer support/ sharing experiences – limited for relatives/family members<br>Impact on expectations and normal milestones for adult and for relatives<br>- Home environment prescribed by adult's needs<br>- Cost of providing for adult and restrictions on earning potential of carers<br><br>THEME 4: **Looking to the Future**<br>Aging care givers<br>Support from services needing fought for year on year<br>Fear for adults life<br>Planning/providing for future needs when no longer able to support<br>- Fear for how adult will cope<br>- Concern for future housing for adult	LIFE QUALITY IN WITH SUPPORTIVE FRIEDNSHIPS OR SPLIT TO TWO THEMES?)		

Table 4. Thematic table showing over-arching Theme 1. 'Family support goes a long way for autistic adults' with four corresponding themes and eleven sub-themes below

Family support goes a long way caring for autistic adults										
Fond admiration		Dependency and independence				Vulnerability and Frustration			Life quality	
Love for autistic adult	Recognition of achievements	Independence of autistic adult	Care and advocacy needs	Impacts of caring on relatives	Supportive relationships	Autistic adult's vulnerabilities	Relatives frustrations with challenges	Misunderstanding autism	Quality of life of autistic adult	Quality of life of relatives

Table 5. Thematic table showing over-arching Theme 2. 'When families recognise they are in need of support from society' with two corresponding themes and six sub-themes below

When families recognise they are in need of support from society					
Engaging with health and social care, education, charities, and informal networks			Looking to the future		
Care providers strengths and weaknesses	Relatives abilities influencing outcomes for autistic adult	Role of informal support networks	Aging caregivers	Fear for future wellbeing of adult	Relatives suggestions for improvements

Example

Figure 1. An example of how an overarching theme and sub-theme linked, with additional underlying groupings and codes

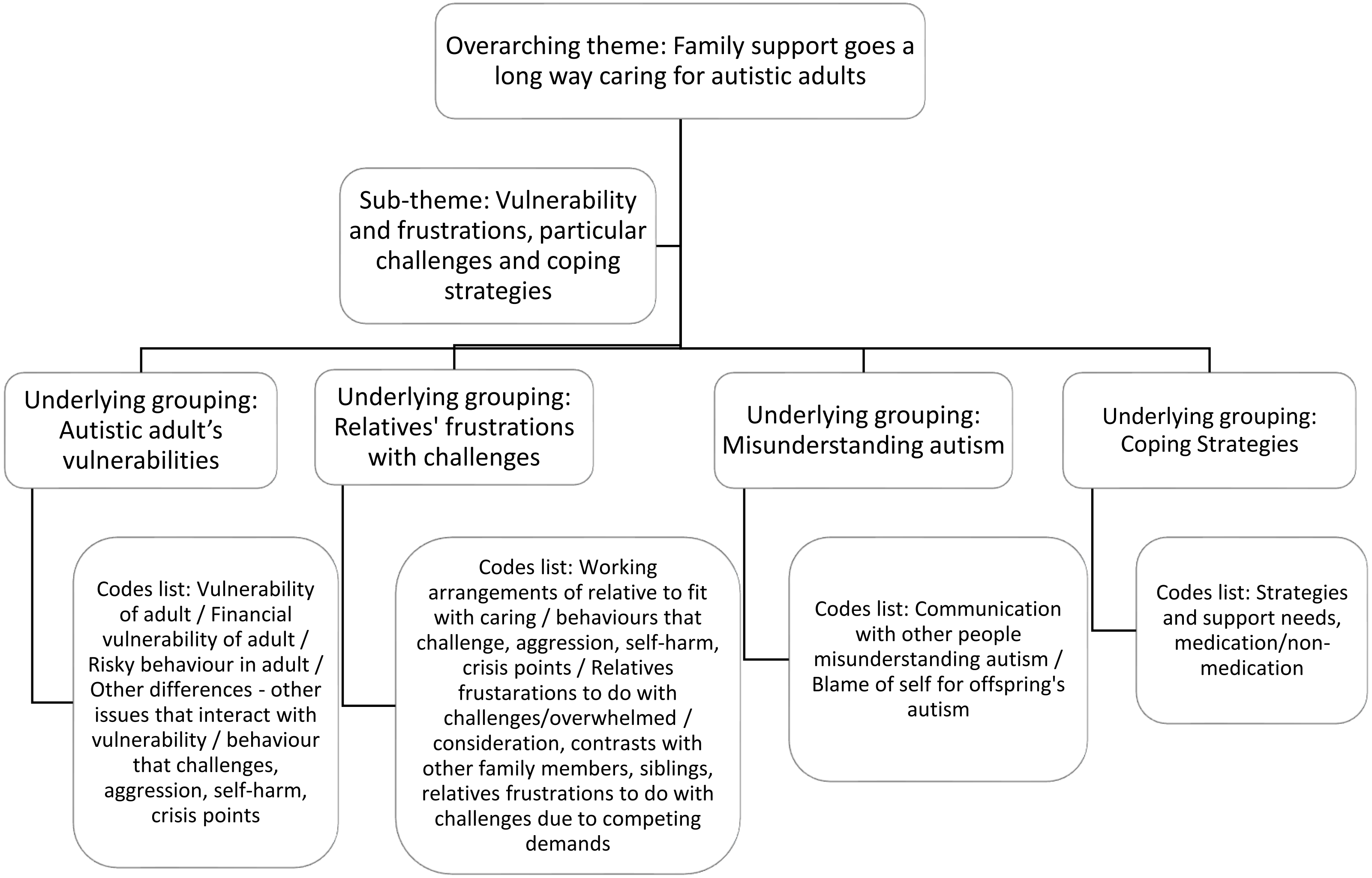

Supplement: S4 File — (PDF) [file pone.0294232.s004.pdf]
