# Supplementary material for: The lived experiences of relatives of autistic adults, and their perceptions of their relationships with autistic adults across multiple age-related transitions and demands: A qualitative interview study with reflexive thematic analysis
Source: PLoS One. 2024 Jan 19;19(1):e0294232. doi: 10.1371/journal.pone.0294232 (PMC10798545; doi:10.1371/journal.pone.0294232)
Supplement: S5 File — (PDF) [file pone.0294232.s005.pdf]

Supporting information 5. Descriptive data metadata*

*Anonymised by removing Pseudonym and shuffling order to protect confidentiality

Relatives

Relatives (Participant) demographics

**Gender**	**Age range**	**Ethnicity**
Female	55-59	White
Female	45-49	White
Male	75-79	White
Female	45-49	White
Female	65-69	White
Male	60-64	White
Female<br>Male	60-64	White
Female	45-49	White
Female	55-59	White
Male	80-84	White
Female<br>Male	60-64	None recorded
Female	60-64	White
Female	50-54	White
Female	30-34	White
Female	40-44	White
Female	60-64	White

**Relatives relationship with autistic adult**
Informally adopted father
Mother
Mother
Mother
Wife
Father

Grandparents (interviewed as a couple)
Mother
Mother
Father
Parents (interviewed as a couple)
Mother
Mother
Mother
Sibling
Mother

**Relatives Qualifications**
No formal qualification
No formal qualification
GCSE / GCE / O-Level / Scottish Higher/ NVQ level 2 / SVQ level 2 or equivalents
GCSE / GCE / O-Level / Scottish Higher/ NVQ level 2 / SVQ level 2 or equivalents
GCSE / GCE / O-Level / Scottish Higher/ NVQ level 2 / SVQ level 2 or equivalents
GCSE / GCE / O-Level / Scottish Higher/ NVQ level 2 / SVQ level 2 or equivalents
A- levels/ NVQ level 3 / SVQ level 3, or equivalents
A- levels/ NVQ level 3 / SVQ level 3, or equivalents
A- levels/ NVQ level 3 / SVQ level 3, or equivalents
A- levels/ NVQ level 3 / SVQ level 3, or equivalents
Certificate of Higher Education
Diploma of Higher Education/Foundation Degree
Bachelors Degree
Bachelors Degree
Postgraduate Degree
Postgraduate Degree

Relatives employment

P/T Admin/Secretarial
P/T. Admin/Secretarial
P/T. Teaching asst
F/T. Sales and customer service
Self-Emp.
F/T Manager/Director/Senior Official
PT Carer to family member
Carer to family member
F/T Carer to family member
Retired
Retired
Retired
Retired
Retired
Retired
Retired
Retired
Retired

Mental health and physical health diagnoses reported for Relatives

Relatives Physical Health Conditions	Relatives Mental Health Conditions
Arthritis, High BP	
Current cancer, arthritis	
Infections	Stress
None	
None	
None	
None	
None	
None	
None	
None	
Recent cancer	
Recent hip replacements	
Registered disabled	
Type 2 Diabetes	Anxiety
Underactive thyroid	Depression
unknown	
unknown	

Autistic Adults

Autistic Adult Related (Delinked for confidentiality)

Gender	Age range	Autism Diagnosis	Learning Disability?
Male	25-29	Asperger	No
Male	55-59	Asperger	Yes
Male	20-24	Atypical Autism	Yes
Male	18-19	Autism	No
Male	20-24	Autism	No
Male	20-24	Asperger	No
Male	55-59	Asperger	No
Male	35-39	Asperger	No
Male	20-24	Asperger	No
Male	18-19	Asperger	No
Female	30-34	Asperger	No
Male	20-24	Autism Spectrum Disorder	No
Female	35-39	Autism Spectrum Disorder	No
Male	25-29	Autism	Yes
Female	30-34	Autism Spectrum Disorder	Yes
Male	20-24	Autism	Yes

Autistic Adults living situation
Independent with wife and children
Independent
Independent
Independent
Independent (family support)
Supported living
Supported living
With parent
With parents
With parents
With parents
With parents
With grandparents

With parent
With parents
With parents

**Autistic Adults Occupation**
Day-care placement
Day-care placement part-time
Employed
Employed part-time
Intermittent employment - unemployed
Intermittent employment - unemployed
Intermittent employment – unemployed
Intermittent employment – unemployed
Specialist college placement
Specialist college placement
Specialist college placement
Student
Student
Unemployed
Unemployed
Unknown

**Adult's education level attained**
Basic School Qualification
Basic School Qualification

Basic School Qualification
Basic School Qualification
HE Bachelor
HE Bachelor
HE Certificate
HE Diploma
High School (GCSE)
High School (GCSE)
High School (GCSE)
High School (GCSE)
No formal qualification
No formal qualification
No formal qualification
No formal qualification

**Adult’s Mental health**	**Neurological conditions**	**Physical health conditions**
Mental health, did not know diagnosis, suicide attempt		
Mental health, depressed, suicidal		
Depression		Diabetes, intercranial hypertension
Recurrent severe depression		
Addictions	Epilepsy	Blood-borne virus

Depression, Anxiety		Type 1 Diabetes, heart problems requiring bypass
Anxiety	Sensory issues and communication difficulties	
Anxiety, OCD	Epilepsy, and communication difficulties	
OCD, Other developmental disorders	Mobility problems, and communication difficulties	
Bipolar disorder		
	Epilepsy, Dyspraxia	intolerance to gluten and dairy
Depression, Anxiety	Dyspraxia. Speech and communication problems.	
Anxiety. ADHD	Dyspraxia	
	Epilepsy	
Anxiety		Peanut allergy, very low BMI, asthma
	Complex communication difficulties related to ASD	
