# Supplementary material for: The lived experiences of relatives of autistic adults, and their perceptions of their relationships with autistic adults across multiple age-related transitions and demands: A qualitative interview study with reflexive thematic analysis
Source: PLoS One. 2024 Jan 19;19(1):e0294232. doi: 10.1371/journal.pone.0294232 (PMC10798545; doi:10.1371/journal.pone.0294232)
Supplement: S1 Checklist — (PDF) [file pone.0294232.s006.pdf]

SRQR Reporting checklist for qualitative study.

Instructions to authors

Complete this checklist by entering the page numbers from your manuscript where readers will find each of the items listed below. SRQR reporting guidelines: O'Brien BC, Harris IB, Beckman TJ, Reed DA, Cook DA. Standards for reporting qualitative research: a synthesis of recommendations. Acad Med. 2014;89(9):1245-1251.

		Reporting Item	Page Number
**Title**			
	#1	Concise description of the nature and topic of the study identifying the study as qualitative or indicating the approach (e.g. ethnography, grounded theory) or data collection methods (e.g. interview, focus group) is recommended	Title<br>✓ Present in Title
**Abstract**			
	#2	Summary of the key elements of the study using the abstract format of the intended publication; typically includes background, purpose, methods, results and conclusions	Abstract ✓<br>Present in Abstract: Background including research questions, methods, main findings, recommendations, conclusions
**Introduction**			
Problem formulation	#3	Description and signifcance of the problem / phenomenon studied: review of relevant theory and empirical work; problem statement	Introduction, Paragraphs 1-3 Para 1-4, pg4&5
Purpose or research question	#4	Purpose of the study and specific objectives or questions	Introduction Para ~~4~~<br>Para 5, pg6
**Methods**			
Qualitative approach and research paradigm	#5	Qualitative approach (e.g. ethnography, grounded theory, case study, phenomenolgy, narrative research) and guiding theory if appropriate; identifying the research paradigm (e.g. postpositivist, constructivist / interpretivist) is also recommended; rationale. The rationale should briefly discuss the justification for choosing that theory, approach, method or technique rather than other options available; the assumptions and limitations implicit in those choices and how those choices influence study conclusions and transferability. As appropriate the rationale for several items might be discussed together.	Introduction – Para ~~4~~ Para 5 pg6<br>~~Methods – Para 1~~
Researcher characteristics and reflexivity	#6	Researchers' characteristics that may influence the research, including personal attributes, qualifications / experience, relationship with participants, assumptions and / or presuppositions; potential or actual interaction between researchers' characteristics and the research questions, approach, methods, results and / or transferability	Methods: Reflexivity ~~Para 1~~ Reflexivity and identity pg11
Context	#7	Setting / site and salient contextual factors; rationale	Methods: setting and site selection Part of Participants pg6
Sampling strategy	#8	How and why research participants, documents, or events were selected; criteria for deciding when no further sampling was necessary (e.g. sampling saturation); rationale	Methods: Participants pg6&7
Ethical issues pertaining to human subjects	#9	Documentation of approval by an appropriate ethics review board and participant consent, or explanation for lack thereof; other confidentiality and data security issues	Ethics – Back Material page & journal system + Methods: Data collection Procedure, pg7&8
Data collection methods	#10	Types of data collected; details of data collection procedures including (as appropriate) start and stop dates of data collection and analysis, iterative process, triangulation of sources / methods, and modification of procedures in response to evolving study findings; rationale	Methods: Data Collection ~~Para 1-3~~ Materials pg7. Dates of data collection in procedure pg7 Procedure and rationale pg7&8
Data collection	#11	Description of instruments (e.g. interview guides,	Methods: Data Materials pg7

instruments and technologies		questionnaires) and devices (e.g. audio recorders) used for data collection; if / how the instruments(s) changed over the course of the study	collection. ~~Para 2~~	
Units of study	#12	Number and relevant characteristics of participants, documents, or events included in the study; level of participation (could be reported in results)	Results: Paragraph 1 and Table 1	Results pg12-16 and Tables 2&3
Data processing	#13	Methods for processing data prior to and during analysis, including transcription, data entry, data management and security, verification of data integrity, data coding, and anonymisation / deidentification of excerpts	Methods: Analysis ~~Paragraph 1~~	Procedure pg8 re transcription and anonymisation Analysis pg9 re coding. Tables show aggregate data to protect anonymity
Data analysis	#14	Process by which inferences, themes, etc. were identified and developed, including the researchers involved in data analysis; usually references a specific paradigm or approach; rationale	Methods: Analysis ~~Paragraph 2~~	Analysis pg9-12
Techniques to enhance trustworthiness	#15	Techniques to enhance trustworthiness and credibility of data analysis (e.g. member checking, audit trail, triangulation); rationale	Methods: Analysis ~~Paragraph 1-2~~	Analysis pg9-12 Supporting information
**Results/findings**				
Syntheses and interpretation	#16	Main findings (e.g. interpretations, inferences, and themes); might include development of a theory or model, or integration with prior research or theory	Results. Paragraph 2-4 + results	Analytic themes pg16 Figure 1. Supporting information
Links to empirical data	#17	Evidence (e.g. quotes, field notes, text excerpts, photographs) to substantiate analytic findings	Results	Analytic themes pg16-31
**Discussion**				
Intergration with prior work, implications, transferability and contribution(s) to the field	#18	Short summary of main findings; explanation of how findings and conclusions connect to, support, elaborate on, or challenge conclusions of earlier scholarship; discussion of scope of application / generalizability; identification of unique contributions(s) to scholarship in a discipline or field	Discussion	Discussion pg32
Limitations	#19	Trustworthiness and limitations of findings	Discussion: Strengths and Limitations	pg34
**Other**				
Conflicts of interest	#20	Potential sources of influence of perceived influence on study conduct and conclusions; how these were managed	Back materials	
Funding	#21	Sources of funding and other support; role of funders in data collection, interpretation and reporting	Back materials	

None The SRQR checklist is distributed with permission of Wolters Kluwer © 2014 by the Association of American Medical Colleges. This checklist can be completed online using https://www.goodreports.org/, a tool made by the EQUATOR Network in collaboration with Penelope.ai
